# Coagulatory active constituents of *Malus pumila* Mill. flowers

**DOI:** 10.1186/s13065-018-0490-6

**Published:** 2018-12-03

**Authors:** Zhenhua Yin, Yong Zhang, Juanjuan Zhang, Jinmei Wang, Wenyi Kang

**Affiliations:** 1 Zhengzhou Key Laboratory of Medicinal Resources Research, Huanghe Science and Technology College, Zhengzhou, 450063 China; 2 Henan Joint International Research Laboratory of Drug Discovery of Small Molecules, Zhengzhou, 450063 China; 3 Joint International Research Laboratory of Food & Medicine Resource Function, Kaifeng, 475004 Henan Province China

**Keywords:** *Malus pumila* Mill, Chemical constituents, Coagulatory activity

## Abstract

**Background:**

The flowers of *Malus pumila* Mill (Rosaceae) is rich in resources, but lack of medicinal value research. Chemical constituents of the same family *M. pumila* have coagulatory activity. Considering the coagulatory activity could be beneficial for various cardiovascular diseases, the aim of this study is to evaluate coagulatory active constituents of *M. pumila* flowers.

**Methods:**

Chemical constituents of *M. pumila* flowers were isolated by various column chromatographies, and their coagulatory activity were evaluated by activated partial thromboplastin time (APTT), prothrombin time (PT), thrombin time (TT) and fibrinogen (FIB) in vitro.

**Results:**

Nine compounds were isolated from *M. pumila* flowers, and identified as kaempferol-3-*O*-*β*-d-glucopyranoside (**1**), kaempferol-7-*O*-*β*-d-glucopyranoside (**2**), kaempferol-3-*O*-*α*-l-arabinofuranoside (**3**), phloridzin (**4**), kaempferol (**5**), phloretin (**6**), *β*-sitosterol (**7**), lupeol (**8**) and pyracanthoside (**9**). Compounds **1–9** were isolated from the flowers for the first time, compounds **1**, **2** and **9** were isolated from the genus for the first time. Compound **2** could significantly shorten APTT, TT and PT, but significantly decrease the content of FIB. Compound **3** could shorten PT. Compound **4** could significantly shorten TT and PT, but significantly decrease the content of FIB. Compound **5** shortened APTT. Compound **6** and **7** could significantly shorten APTT and PT. Compound **9** was able to prolong TT and decrease the content of FIB, but shorten PT.

**Conclusions:**

Compounds **2–7** possessed significant procoagulant activity in vitro, compound **9** had anticoagulant activity in vitro, which showed coagulation potential of compounds from *M. pumila* flowers, as a new source of bioactive molecules for therapeutic purposes.

## Background

Thrombosis involves local blood clotting of the vascular system, often leading to serious health-related diseases such as heart attacks and strokes. Risk factors for thrombosis include hyperlipidemia, hyperglycemia, hypertension and cancer. These thrombotic diseases have become the leading cause of death, and incidence is increasing annually [1, 2]. In the past few decades, drugs, including heparin and warfarin, have been used to prevent and treat thrombosis, but heparin is prone to spontaneous bleeding, and manifested by various mucosal hemorrhages, joint bleeding and wound bleeding. In addition, heparin-induced thrombocytopenia is a serious complication of heparin therapy [3, 4]. Therefore, these disadvantages have necessitated a field of research aimed at discovering novel anti-thrombotic and anti-coagulant agents with fewer side effects than heparin.

*Malus pumila* Mill, belonging to the family Rosaceae, has been widely cultivated in around the world for centuries [5]. At present, the study of *Malus pumila* focused on its fruits, peels, leaves and branches. Previous research indicated that its peels contained triterpenoids, flavonoids, phenols and other components such as alkyl alcohols, and exhibited more potent antioxidant and antiproliferative activities [6–8]. The fruits of this plants contained flavonoids, polyphenols, glycosides, triterpenoids, steroids and fatty acid esters, and exhibited antioxidant activity [9–13].The leaf of apple has been reported to contain high levels of polyphenols, flavonoids, and exhibit great in vitro antioxidant activity and protective effect in reserpine-induced gastric ulcer in mice [14, 15]. However, chemical constituents and pharmacological effects of *M. pumila* flowers are still uncertain without a clear theoretical evidence.

The bioactive compounds of *M. pumila* flowers, may be responsible for coagulation activity, but not clear. The objective of this research was to isolate and identify the bioactive compounds of *M. pumila* flowers with potent coagulation activity, which might expand the possibility to find better coagulation drug.

## Methods

### Plant collection

Fresh flowers of *Malus pumila* were collected from Henan University Medicinal Botanical Garden in Henan Province (China) in April 2016. The specimens were authenticated and identified by Prof. Changqin Li (Henan University) according to Flora of Henan, and a voucher (specimen No: 20160410) was been deposited in the Herbarium of Huanghe Science and Technology College.

### Extraction and isolation

The air-dried flowers of *M. pumila* (700 g) were degreased repeatedly (3 times) with petroleum ether at room temperature, 3 days each time. After petroleum ether was evaporated, the residue was extracted 3 times with 70% ethanol. The ethanol extract was concentrated under reduced pressure to give ethanol extract. The extract was evenly dispersed with an appropriate amount of water, followed by extraction with petroleum ether, ethyl acetate and *n*-butanol. The solvent was concentrated under reduced pressure to obtain petroleum ether extract (12.0074 g), EtOAc extract (26.6719 g) and *n*-butanol extract (59.1956 g).

The EtOAc extract was dissolved in 70% ethanol, adsorbed and loaded with D101 macroporous resin, and allowed to stand overnight, followed by gradient elution (H_2_O: ethanol 100%, 70%, 40%, 10%). The solvents were concentrated under reduced pressure to get water extract, 30%, 60% and 90% ethanol fractions.

The first and second column volumes of 60% ethanol fraction were combined and applied on a silica gel H using a gradient elution of CH_2_Cl_2_–acetone (10: 1–6:1), and then further separated by column chromatography over silica gel H by a gradient elution of CH_2_Cl_2_–MeOH (10: 1–0:1) to afford three fractions (Fr1, Fr2 and Fr3). Fr1 was chromatographed on silica gel by gradient elution with a CH_2_Cl_2_–acetone (4: 1–2:1) to afford two subfractions (Fr1.1 and Fr1.2). Fr1.1 (98.7 mg) was further subjected to Sephadex LH-20 (MeOH), then purified using a Gilson prepared liquid phase C18 column eluted with Acetonitrile–water containing 0.1% formic acid in water (35:65–60:40), and further purified using a Agilent SB-Phenyl column eluted with MeOH–water containing 0.1% formic acid in water (60:40–90:10; 50:50) to yield compound **1** (23.0 mg) and compound **2** (5.3 mg). Fr1.2 was subjected to Sephadex LH-20 (MeOH), and further purified using a Gilson prepared liquid phase C18 column eluted with Acetonitrile–water containing 0.1% formic acid in water (30:70–45:55) to yield compound **3** (20.3 mg). Fr3 was subjected to Sephadex LH-20 (MeOH), and then further purified by twice preparative liquid phases to yield compound **4** (200.3 mg).

The third column volume of 60% ethanol extract was subjected to silica gel (200–300 mesh) column chromatography and gradient eluted with a CH_2_Cl_2_–acetone (30: 1–8:1), and then was further chromatographed on silica gel H by gradient elution with a CH_2_Cl_2_–MeOH (7: 1–1:1) to afford two fractions (Fr1 and Fr 2), respectively. Fr1 was separated by column chromatography over silica gel H by elution with a CH_2_Cl_2_–acetone (10:1) to yield compound **5** (10.2 mg), and Fr 2 was applied on silica gel H using elution with a CH_2_Cl_2_–acetone (10:1) containing 30 μL formic acid to afford compound **6** (4.7 mg).

The first and second column volumes of 90% ethanol extract were combined together, and then was chromatographed on silica gel H by gradient elution with a CH_2_Cl_2_–acetone (40: 1–10:1), and was further purified on Sephadex LH-20 (CH_2_Cl_2_: MeOH = 1: 1) to yield compound **7** (5.0 mg). The third column volume of 90% ethanol extract was exposed to column chromatography on silica gel H eluted with CH_2_Cl_2_-EtOAc (20:1), then separated on Sephadex-LH 20 (CH_2_Cl_2_:MeOH = 1: 1), and further purified by a semi-prepared high performance liquid phase reversed phase C18 column eluted with acetonitrile–water containing 0.1% formic acid in water (40:60–70:30) to yield compound **8** (4.7 mg).

The *n*-BuOH extract was subjected to silica gel (200–300 mesh) column chromatography and gradient eluted with a CH_2_Cl_2_–MeOH (100: 1–1:1) to give six fractions, where the second fraction was subjected to silica gel H eluted with a CH_2_Cl_2_-MeOH gradient (15:1–5:1), and then was subjected to Sephadex-LH 20 (MeOH) to yield compound **9** (2.4 mg). In conclusion, assigned the compound numberings for all the isolated compounds (**1**–**9**).

### Coagulation time test in vitro

The coagulation activity of compounds **1–7** and **9** were evaluated by activated partial thromboplastin time (APTT), thrombin time (TT), prothrombin time (PT), and fibrinogen (FIB) assays in vitro.

Blood samples were drawn from the auricular veins of rabbits, and the method of blood samples preparation was carried out according to Wang et al. [16]. APTT and PT were determined using the method reported by Chen et al. [17, 18]. Briefly, for APTT assays in vitro, 50 μL serum was incubated with 25 μL samples and 50 μL APTT assay reagent for 5 min at 37 °C. The Clotting times were immediately recorded after the addition of 25 mM CaCl_2_ (100 μL). For PT assays in vitro, 50 μL serum was incubated with 25 μL samples for 3 min at 37 °C, and then PT assay reagent (50 μL), which had been incubated at 37 °C for 10 min, was added to immediately recorded clotting time. Determinations of TT and FIB were performed according to the manufacturer’s recommendations (Shanghai Sun Biotech Co., Ltd, China). All coagulation assays were performed in triplicates. Breviscapine (13.33 mg/mL) and Yunnanbaiyao (40 mg/mL) were used as positive controls, and the blank solvent (anhydrous ethanol: 1,2-propylene glycol: physiological saline = 1: 1: 3, volume ratio) were used as negative controls. The concentrations of compounds **1–7** and **9** were 5 mg/mL. PT, APTT, TT and FIB tests were conducted by a Semi-Automated Coagulation Analyzer (CPC Diagnostics Pvt. Ltd, India).

The results were expressed as mean ± SD for three independent experiments. Numerical statistics were performed using SPSS19.0 software with single factor analysis of variance (ANOVA One-Way) to determine the difference.

## Results and discussion

**Fig. 1:**
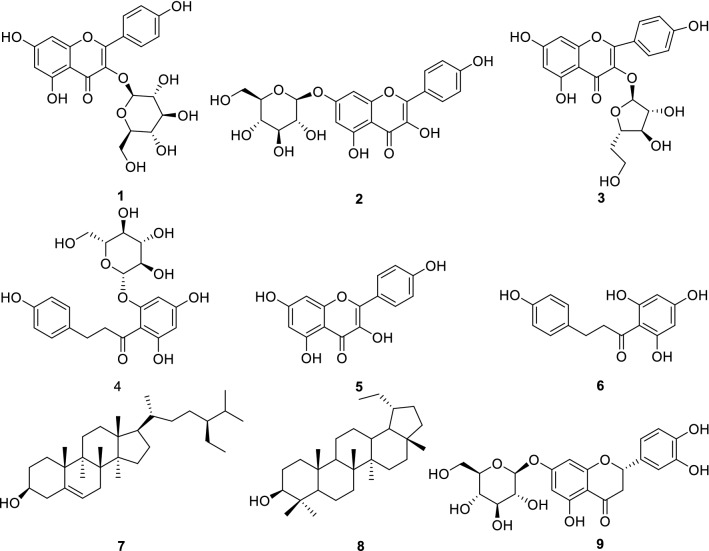
Structures of compounds **1**–**9**

A total of nine compounds (**1**–**9**), were isolated from *M. pumila* flowers for the first time. Among them, compounds **1**, **2** and **9** were isolated from the genus for the first time (Fig. 1). The chemical structures of these compounds were identified as kaempferol-3-*O*-*β*-d-glucopyranoside (**1**) [19], kaempferol-7-*O*-*β*-d-glucopyranoside (**2**) [20], kaempferol-3-*O*-*α*-l-arabinofuranoside (**3**) [21], phloridzin (**4**) [22], kaempferol (**5**) [23], phloretin (**6**) [24], *β*-sitosterol (**7**) [25], lupeol (**8**) [26], pyracanthoside (**9**) [27].

Kaempferol-3-*O*-*β*-d-glucopyranoside (**1**): yellow needle crystal (MeOH), C_21_H_20_O_11_, ESI–MS *m/z*: 431[M-H]^+^. ^1^H-NMR (DMSO-*d*_6_, 400 MHz) *δ*: 12.61 (1H, s, 5-OH), 8.03 (2H, d, *J *= 8.0 Hz, H-2′,6′), 6.88 (2H, d, *J *= 8.0 Hz, H-3′,5′), 6.43 (1H, s, H-8), 6.21 (1H, s, H-6), 5.45 (1H, d, *J *= 8.0 Hz, H-1″). ^13^C-NMR (DMSO-*d*_6_, 100 MHz) *δ*: 156.3 (C-2), 133.3 (C-3), 177.5 (C-4), 161.3 (C-5), 98.8 (C-6), 164.3 (C-7), 93.7 (C-8), 156.5 (C-9), 104.0 (C-10), 121.0 (C-1′), 130.9 (C-2′), 115.2 (C-3′), 160.0 (C-4′), 115.2 (C-5′), 130.9 (C-6′), 101.0 (C-1′′), 74.3 (C-2′′), 76.5 (C-3′′), 70.0 (C-4′′), 77.5 (C-5′′), 60.9 (C-6′′).

Kaempferol-7-*O*-*β*-*D*-glucopyranoside (**2**): yellow powder (MeOH), C_21_H_20_O_11_, ESI–MS *m/z*: 449[M + H]^+^. ^1^H-NMR (DMSO-*d*_6_, 400 MHz) *δ*: 8.05 (2H, d, *J *= 8.0 Hz, H-2′,6′), 6.92 (2H, d, *J *= 8.0 Hz, H-3′,5′), 6.75 (1H, s, H-8), 6.41 (1H, s, H-6), 5.10 (1H, s, H-1″). ^13^C-NMR (DMSO-*d*_6_, 100 MHz) *δ*: 147.4 (C-2), 135.5 (C-3), 175.7 (C-4), 160.3 (C-5), 98.8 (C-6), 162.3 (C-7), 94.3 (C-8), 155.8 (C-9), 104.5 (C-10), 121.3 (C-1′), 129.8 (C-2′), 115.5 (C-3′), 160.0 (C-4′), 115.5 (C-5′), 129.8 (C-6′), 100.1 (C-1′′), 73.2 (C-2′′), 77.2 (C-3′′), 69.4 (C-4′′), 76.5 (C-5′′), 60.7 (C-6′′).

Kaempferol-3-*O*-*α*-l-arabinofuranoside (**3**): yellow powder (MeOH), C_20_H_18_O_10_, ESI–MS *m/z*: 419[M + H]^+^. ^1^H-NMR (DMSO-*d*_6_, 400 MHz) *δ*: 12.63 (1H, s, 5-OH), 8.02 (2H, d, *J *= 8.0 Hz, H-2′,6′), 6.89 (2H, d, *J *= 8.0 Hz, H-3′,5′), 6.45 (1H, s, H-8), 6.21 (1H, s, H-6), 5.63 (1H, s, H-1″). ^13^C-NMR (DMSO-*d*_6_, 100 MHz) *δ*: 156.4 (C-2), 133.5 (C-3), 177.7 (C-4), 161.3 (C-5), 98.8 (C-6), 164.3 (C-7), 93.4 (C-8), 156.9 (C-9), 104.1 (C-10), 120.8 (C-1′), 130.8 (C-2′), 115.5 (C-3′), 160.0 (C-4′), 115.5 (C-5′), 130.8 (C-6′), 108.1 (C-1′′), 82.2 (C-2′′), 77.2 (C-3′′), 86.4 (C-4′′), 61.0 (C-5′′).

Phloridzin (**4**): white needle crystal (MeOH), mp. 168–169 °C, C_21_H_24_O_10_, ESI–MS *m/z*: 437[M + H]^+^. ^1^H-NMR (DMSO-*d*_*6*_, 400 MHz) *δ*: 7.03 (2H, d, *J *= 8.0 Hz, H-3, 5), 6.63 (2H, d, *J *= 8.0 Hz, H-2, 6), 6.13 (1H, s, H-5′), 5.93 (1H, d, *J *= 4.0 Hz, H-3′), 5.33 (1H, d, *J *= 4.0 Hz, OH-4′′), 5.19 (1H, d, *J *= 4.0 Hz, OH-3′′), 5.09 (1H, d, *J *= 4.0 Hz, OH-2′′), 4.93 (1H, d, *J *= 8.0 Hz, H-1′′), 4.63 (1H, t, *J *= 8.0, 4.0 Hz, OH-6′′), 3.71 (1H, m, H-6′′), 3.19 (1H, t, *J *= 8.4 Hz, H-2′′), 2.78 (2H, t, *J *= 8.0, 8.0 Hz, H-*β*). ^13^C-NMR (DMSO-*d*_*6*_, 100 MHz) *δ*: 29.0 (C-*β*), 45.0 (C-*α*), 204.7 (C = O), 131.6 (C-1), 129.2 (C-2, 6), 115.0 (C-3, 5), 155.3 (C-4), 105.2 (C-1′), 165.4 (C-2′), 96.9 (C-3′), 164.6 (C-4′), 94.4 (C-5′), 160.8 (C-6′), 100.8 (C-1′′), 73.2 (C-2′′), 77.3 (C-3′′), 69.5 (C-4′′), 76.7 (C-5′′), 60.6 (C-6′′).

Kaempferol (**5**): yellow powder (MeOH), C_15_H_10_O_6_, ESI–MS *m/z*: 287[M + H]^+^. ^1^H-NMR (MeOH, 400 MHz) *δ*: 8.08 (2H, d, *J *= 6.4 Hz, H-2′,6′), 6.89 (2H, d, *J *= 7.6 Hz, H-3′,5′), 6.39 (1H, s, H-8), 6.18 (1H, s, H-6). ^13^C-NMR (100 MHz, MeOH) *δ*: 146.8 (C-2), 135.6 (C-3), 175.9 (C-4), 156.2 (C-5),99.3 (C-6), 162.5 (C-7), 94.4 (C-8), 162.5 (C-9), 103.1 (C-10), 123.9 (C-l′), 130.6 (C-2′), 116.3 (C-3′), 160.4 (C-4′), l16.3 (C-5′), 130.6 (C-6′).

Phloretin (**6**): white powder (MeOH–water), C_15_H_14_O_5_. ^1^H-NMR (DMSO-*d*_6_, 400 MHz) *δ*: 7.02 (2H, d, *J *= 7.6 Hz, H-2, 6), 6.66 (2H, d, *J *= 7.6 Hz, H-3, 5), 5.76 (2H, brs, H-3′,5′), 3.21 (2H, t, *J *= 7.6 Hz, H-*α*), 2.75(2H, t, *J *= 7.4 Hz, H-*β*). ^13^C-NMR (100 MHz, DMSO-*d*_6_) *δ*: 132.2 (C-1), 130.1 (C-2, 6), 115.5 (C-3, 5), 155.9 (C-4), 204.4 (C = O), 104.2 (C-1′), 165.4 (C-2′, 6′), 95.1 (C-3′, 5′), 165.9 (C-4′), 45.8 (C-*α*), 30.1(C-*β*).

*β*-Sitosterol (**7**): white needle crystal (acetone), C_29_H_50_O, mp 136–137 °C, EI-MS *m/z*: 414.4[M]^+^. ^1^H-NMR (CDCl_3_,400 MHz) *δ*: 5.36 (1H, brs, H-6), 3.52 (1H, m, H-3*α*), 0.68 (3H, s, CH_3_-18), 0.80 (3H, s, CH_3_-27), 0.82 (3H, d, *J *= 8 Hz, CH_3_-26), 0.86 (3H, s, CH_3_-29), 0.93 (3H, d, *J *= 8 Hz, CH_3_-21), 1.01 (3H, s, CH_3_-19).

Lupeol (**8**): white needle crystal (chloroform-acetone), mp 214–217 °C. C_30_H_50_O. EI-MS *m/z*: 426[M]^+^. ^1^H-NMR (CDCl_3_, 400 MHz) *δ*: 4.69 (1H, brs, H-29), 4.57 (1H, brs, H-29), 3.19 (1H, dd, *J *= 11.2, 5.2 Hz, H-3), 2.38 (1H, m, H-19), 1.68 (3H, s, H-30), 1.03 (3H, s, H-26), 0.97 (3H, s, H-23), 0.95 (3H, s, H-27), 0.83 (3H, s, H-25), 0.79 (3H, s, H-28), 0.76 (3H, s, H-24). ^13^C-NMR (CDCl_3_, 100 MHz) *δ*: 38.9 (C-1), 27.7 (C-2), 79.2 (C-3), 39.1 (C-4), 55.5 (C-5), 18.5 (C-6), 34.5 (C-7), 41.1 (C-8), 50.7 (C-9), 37.4 (C-10), 21.2 (C-11), 25.4 (C-12), 38.3 (C-13), 43.0 (C-14), 27.6 (C-15), 35.8 (C-16), 43.2 (C-17), 48.2 (C-18), 48.6 (C-19), 151.1 (C-20), 30.1 (C-21), 40.2 (C-22), 28.2 (C-23), 15.5 (C-24), 16.3 (C-25), 16.2 (C-26), 14.7 (C-27), 18.2 (C-28), 109.5 (C-29), 19.5 (C-30).

Pyracanthoside (**9**): light yellow powder (MeOH), C_21_H_22_O_11_, ESI–MS *m/z*: 449[M-H]^−^, 287[M-H-Glc]^−^. ^1^H-NMR (MeOH, 400 MHz) *δ*: 2.62 (1H, d, *J *= 5.8 Hz, H-3*α*), 2.96 (1H, d, *J *= 2.6 Hz, H-3*β*), 4.75 (1H, d, *J *= 6.4 Hz, H-1′′), 5.32 (1H, d, *J *= 5.8 Hz, H-3*α*), 6.38 (1H, s, H-8), 6.85 (1H, d, *J *= 9.4 Hz, H-5′), 7.46 (1H, dd, *J *= 1.8, 9.4 Hz, H-6′), 7.50 (1H, d, *J *= 1.8 Hz, H-2′). ^13^C-NMR (100 MHz, MeOH) *δ*: 78.8 (C-2), 42.2 (C-3), 197.2 (C-4), 163.0 (C-5),96.5 (C-6), 165.3 (C-7), 95.5 (C-8), 162.7 (C-9), 103.3 (C-10), 129.2 (C-l′), 114.5 (C-2′), 145.8 (C-3′), 145.2 (C-4′), l15.4 (C-5′), 118.2 (C-6′), 99. 6 (C-1′′), 73.0 (C-2′′), 76.3 (C-3′′), 69.5 (C-4′′), 77.1 (C-5′′), 60.6 (C-6′′).

Flavonoids, steroids and terpenes were isolated from the flowers of *M. pumila*. Several secondary metabolites from plants that present many important biological activities, such as anticoagulant and antioxidant [28], some play a important role in triggering cardiovascular activities. Cao et al. [29] reported that flavonoids such as acacetin and tilianin exhibited significant anticoagulant activity through prolonging PT, APTT, TT and reducing FIB content, and Xie et al. [25] found terpenes such as ursolic acid, oleanolic acid and suavissimoside R1 exhibited anticoagulant activity in vitro. These findings prompted us to study the coagulatory active constituents from flowers of *M. pumila*, seeking new therapeutic purposes for this plant.

**Fig. 2:**
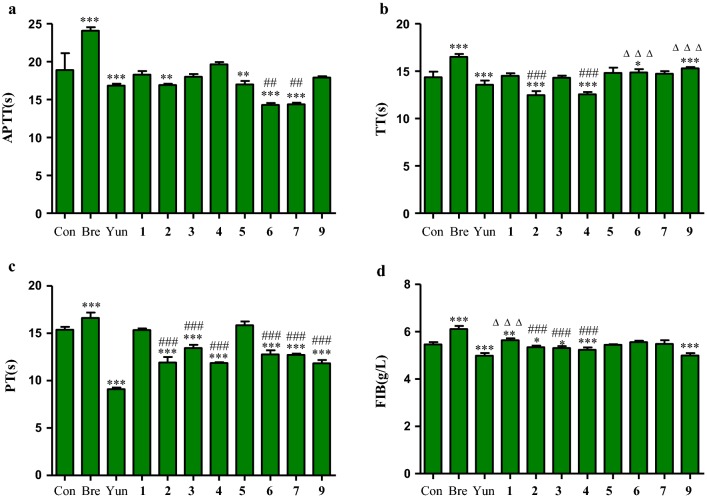
Effects of compounds **1–7** and **9** on plasma coagulation parameters in vitro (**a** APPT; **b** PT; **c** TT; **d** FIB. n = 6). Compared with control group (Con), ^***^p < 0.001 < ^**^p < 0.01 < ^*^p < 0.05; Compared with Yunnan Baiyao (Yun), ^###^*p *< 0.001 < ^##^*p *< 0.01 < ^#^*p *< 0.05; Compared with breviscapine (Bre), ^△△△^*p *< 0.001 < ^△△^*p *< 0.01 < ^△^*p *< 0.05

The result of coagulation activity of compounds **1–7** and **9** isolated from flowers were showed in Fig. 2a. In APTT test, compared with blank group, compounds **2** and **5–7** were able to significantly shorten the clotting time (*p *< 0.001 or *p *< 0.01), and the effect of compounds **6** and **7** was significantly better than that of Yunnanbaiyao as positive control (*p *< 0.01), demonstrating their procoagulant activity.

As can be observed in Fig. 2b, in TT test, compared with blank group, compounds **2** and **4** could be significantly shorten the clotting time (*p *< 0.001), and was significantly better than Yunnanbaiyao (*p *< 0.001), while compounds **6** and **9** significantly prolonged the clotting time compared with blank group (*p *< 0.001 or *p *< 0.05), and the effect was weaker than breviscapine as positive control, and had statistical significance (*p *< 0.001).

As can be observed in Fig. 2c, in PT test, compounds **2–4**, **6–7** and **9** could significantly shorten clotting time compared with blank group (*p *< 0.001), and the effect was weaker than Yunnanbaiyao, and had statistical significance (*p *< 0.001).

As can be observed in Fig. 2d, in FIB test, compared with blank group, compounds **2–4** and **9** could significantly decrease the FIB content (*p *< 0.001 or *p *< 0.05), and the effect of compounds **2–4** was significantly weaker than Yunnanbaiyao (*p *< 0.001), compound **9** was equal to that of Yunnanbaiyao (*p *> 0.05), compound **1** could significantly increase the FIB content compared with blank group (*p *< 0.01), and was significantly weaker than breviscapine (*p *< 0.001).

Normally, 12 blood coagulatory factors are sequentially combined with coagulation process, which include the intrinsic and/or common pathway and extrinsic pathway [30]. In clinical tests of blood coagulation, several well-established analyses are used to indicate coagulation activity including APTT, PT, TT and FIB. Normally, PT is used to evaluate the overall efficiency of extrinsic clotting pathway, a prolonged PT indicates a deficiency in coagulation factors V, VII and X. On the other hand, APTT is a test of the intrinsic clotting activity, a prolonged APTT usually represents a deficiency in factors VIII, IX, XI, XII and VonWillebrand’s factor. Whereas prolongation of TT indicates inhibition of thrombin activity or polymerization, FIB mainly reflects the content of fibrinogen [25, 31, 32]. In this study, compound **2** could significantly shorten APTT, TT and PT, but significantly decrease the content of FIB, it could therefore be speculated that its procoagulant activity might be related to the changes of coagulation factors in both extrinsic and intrinsic clotting pathways. For compound **3**, shortening PT demonstrated inhibition of the extrinsic pathway. Compound **4** could significantly shorten TT and PT, but significantly decrease the content of FIB, demonstrating the changes of coagulation factor activity in exogenous coagulation pathways caused its procoagulant activity. For compound **5**, shortening APTT demonstrated inhibition of the intrinsic clotting pathway. Compound **6** and **7** could significantly shorten APTT and PT, demonstrating their procoagulant activity might result from altered activity of coagulation factors in both extrinsic and intrinsic clotting pathways. Compound **9** was able to prolong TT and decrease the content of FIB, but shorten PT, demonstrating its anticoagulant activity might be related to activation of thrombin activity.

In conclusion, the presented results showed that compounds **2–7** had significant beneficial effects as a procoagulant agent, compound **9** had significant beneficial effects as an anticoagulant agent. Thus, this study showed the potential of *M. pumila* flowers as a new source of bioactive molecules for therapeutic purposes. Up to now, many scholars have paid attention to the structure–activity relationship of active natural products and modified their structures, for example, Reddy et al. [33–35] had modified the natural products with antioxidant properties and antiproliferative activities, which would guide us to further study on compounds **2–7** and **9**.

## Conclusion

Nine compounds were isolated from the flowers of *M. pumila*, and identified as kaempferol-3-*O*-*β*-d-glucopyranoside, kaempferol-7-*O*-*β*-d-glucopyranoside, kaempferol-3-*O*-*α*-l-arabinofuranoside, phloridzin, kaempferol, phloretin, *β*-sitosterol, lupeol and pyracanthoside. All compounds were isolated from the flowers for the first time, compounds **1**, **2** and **9** were isolated from the genus for the first time. Compounds **2–7** had procoagulant activity, compound **9** had anticoagulant activity. Since compounds with procoagulant or anticoagulant that could be used in current medicine for treatment of various cardiovascular diseases, we suggested that the flowers of *M. pumila* showed promising potential as a future therapeutic agent. Thus, the results presented herein might provide scientific evidence, at least, for the widespread use of this plant in complementary and alternative drugs and to demonstrate its potential as a new source of bioactive molecules for therapeutic purposes.

## Abbreviations

ATPP: activated partial thromboplastin time
TT: thrombin time
PT: prothrombin time
FIB: fbrinogen
Con: control group
Yun: Yunnan Baiyao
Bre: breviscapine (Bre)
